# In Middle-Aged Adults, Cognitive Performance Improves After One Year of Auditory Rehabilitation with a Cochlear Implant

**DOI:** 10.3390/brainsci16010022

**Published:** 2025-12-24

**Authors:** Jaron Zuberbier, Agnieszka J. Szczepek, Heidi Olze

**Affiliations:** 1 Department of Otorhinolaryngology, Head and Neck Surgery, Charité—Universitätsmedizin Berlin, Corporate Member of Freie Universität Berlin and Humboldt Universität zu Berlin, 10117 Berlin, Germany; jaron.zuberbier@charite.de (J.Z.);; 2 Faculty of Medicine and Health Sciences, University of Zielona Góra, 65-046 Zielona Góra, Poland

**Keywords:** cochlear implant, hearing impairment, dementia, tinnitus

## Abstract

**Background/Objectives**: Hearing impairment in middle-aged adults is a significant, modifiable risk factor for cognitive decline and dementia, and therapy with hearing aids or cochlear implants has been suggested to reduce this risk. However, most research on auditory rehabilitation and cognition has focused on older adults, and evidence regarding cognitive outcomes in middle-aged adults remains scarce despite this group being identified as critical for dementia prevention. Thus, this study aimed to assess cognitive skills in middle-aged hearing-impaired individuals 1 year after receiving a cochlear implant (CI) as part of auditory rehabilitation. **Methods**: Thirty-two patients with a mean age of 52.4 were enrolled in a prospective pre-post study. Hearing was tested using the Freiburg Monosyllable Test (FS) and the Oldenburg Inventory (OI). Cognitive performance was assessed using the WAIS-IV, operationalized through the Working Memory Index (Digit Span, Arithmetic) and Processing Speed Index (Symbol Search, Coding). Quality of life was assessed with the NCIQ, tinnitus-related distress with the Tinnitus Questionnaire (TQ), and depressive symptoms with the ADS-L. **Results**: After one year, speech intelligibility (FS) improved from a median of 0 to 70.0 (Wilcoxon Z = −4.864, *p* < 0.001, r = −0.61), and subjective hearing from a median of 2.55 to 3.18 (Wilcoxon Z = −3.072, *p* = 0.002). The NCIQ score increased from 52.3 to 60.6 (Z = −3.899, *p* < 0.001), and tinnitus-related distress decreased from 25 to 21 (Wilcoxon Z = −2.209, *p* = 0.027). Depressive symptoms declined numerically, although this change did not reach statistical significance. Working memory improved from 82.0 to 89.0 (Wilcoxon Z = −4.090, *p* < 0.001), and processing speed from 89.5 to 95.5 (Wilcoxon Z = −2.533, *p* = 0.011). Before CI, WMI and PSI showed a strong correlation (ρ = 0.533, *p* = 0.002), and WMI correlated moderately with education level (ρ = 0.452, *p* = 0.012). One year after CI, correlations strengthened between PSI and NCIQ (ρ = 0.510, *p* = 0.006), PSI and OI (ρ = 0.400, *p* = 0.039), and WMI and TQ (ρ = –0.459, *p* = 0.021), indicating emerging associations between cognitive outcomes and auditory or psychosocial measures. **Conclusions**: One year of CI-based auditory rehabilitation improves auditory function, quality of life, tinnitus distress, and—critically—working memory and processing speed in middle-aged adults. These findings address a previously unfilled research gap and support the relevance of CIs for preserving cognitive health during midlife.

## 1. Introduction

Hearing is an essential component of human communication, and its impairment is among the most common communication disorders, significantly reducing the quality of life of affected individuals [1]. The WHO estimates that the number of people with hearing loss will reach 2.5 billion in 2050 [2]. Hearing loss may arise from hereditary, conductive, sensorineural, or central causes, and treatment options range from cerumen removal to hearing aids and neuroprosthetic interventions such as cochlear implantation [3]. Dementia, another highly prevalent disorder with substantial global burden, affected approximately 50 million people in 2017 [4] and is characterized by progressive cognitive decline impairing independent daily functioning [5]. According to WHO projections, this number will rise to 150 million by 2050. Although dementia has been known for millennia [6], its mechanisms and modifiable determinants have only recently been extensively elucidated [5].

Dementia and hearing loss are both strongly associated with aging [7]. As populations age worldwide, these conditions increasingly contribute to public health challenges [8,9]. In 2017, Livingston et al. published an influential evidence-based report identifying modifiable risk factors for dementia, highlighting untreated hearing loss during midlife (ages 45–65) as a major contributor to later cognitive decline [10]. The Lancet Commission reports from 2020 and 2024 reaffirm this finding, highlighting that nearly half of dementia cases may be prevented or delayed through targeted strategies. Midlife hearing impairment remains the most significant modifiable risk factor identified [11,12].

Since hearing loss was recognized as a risk factor for cognitive decline and dementia, research has increasingly focused on how auditory rehabilitation—via hearing aids or cochlear implants (CIs)—affects cognitive outcomes. A recent meta-analysis demonstrated that the use of hearing devices is associated with a 19% reduction in the risk of cognitive decline [13]. However, nearly all studies investigating cognitive outcomes after CI have focused on older adults, leaving adults aged 45–65—the age group most strongly linked to dementia prevention—substantially underrepresented.

Only two previous studies have included individuals younger than 65 years. Mosnier et al. evaluated 100 CI recipients using tests such as the MMSE, TMT-B, and Digit Symbol Coding, and reported cognitive improvement only in the youngest subgroup (60–64 years) [14]. Calvino et al. studied 28 CI candidates, aged ≤ 60 and ≥61 years, using the RBANS-H and reported postoperative cognitive improvement in both groups [15]. However, neither study specifically examined the 45–65-year age range, nor did they employ the WAIS-IV, the clinical gold standard for measuring working memory and processing speed [16]. Furthermore, both studies used heterogeneous cognitive batteries that limit comparability across domains relevant to auditory–cognitive interactions. As summarized in Table 1, existing studies targeting the middle-aged age group vary widely in age ranges, cognitive measures, and methodologies, leaving the cognitive effects of cochlear implantation in middle-aged adults not well studied.

Thus, a significant research gap persists: it remains unknown whether CI-based auditory rehabilitation improves cognition—specifically, working memory and processing speed—in middle-aged adults, the age group in which hearing loss is most strongly associated with increased dementia risk. Therefore, the present study investigates whether one year of CI-based auditory rehabilitation enhances working memory and processing speed in middle-aged adults. Because cognitive performance is intertwined with psychosocial and auditory functioning, subjective auditory performance, tinnitus-related distress, depressive symptoms, and health-related quality of life were also assessed to provide a comprehensive evaluation of rehabilitation effects.

## 2. Materials and Methods

This prospective longitudinal study examined adult patients scheduled to receive cochlear implants (CIs) at two time points: before implantation (baseline) and after 12 months of CI-based auditory rehabilitation. Ethical approval was obtained from the Ethics Committee of Charité Universitätsmedizin Berlin under the reference number (EA2/030/13). All participating patients provided signed informed consent for the study. The data collection period lasted from 2018 to 2021.

### 2.1. Study Population and Inclusion Criteria

The study included 32 patients (ages 32 to 65; see Table 2) who consecutively presented at a tertiary healthcare facility and met the medical criteria for unilateral cochlear implantation, as outlined in the German guidelines [17]. The a priori sample size calculated using power analysis (one-sample mean) was 29 subjects, with an effect size d = 0.7, alpha = 0.05, and power = 0.95. The sample was heterogeneous with respect to the cause of hearing loss and laterality. The indications for CI were evaluated in accordance with national guidelines [18]. Of the 32 patients included, 22 used hearing aids in the ear scheduled for cochlear implantation during their pre-surgical diagnostic appointments. Ten patients (2 AHL, 7 SSD, 1 DSD) either discontinued hearing aids because they found no benefit (AHL, DSD) or were ineligible for use due to the severity of their hearing loss (SSD). The analysis grouped individuals with unilateral, bilateral, and asymmetric hearing loss into a single cohort. This decision reflects the study’s primary objective: to examine cognitive outcomes rather than the specific cause or side of the hearing impairment. Evidence from the CI literature indicates that cognitive performance in hearing-impaired adults is primarily affected by auditory deprivation, increased listening effort, and reduced auditory input, regardless of whether the hearing loss is unilateral or bilateral [19,20,21]. All participants met the same candidacy criteria for cochlear implantation, underwent the same rehabilitation protocol, and received the same cognitive assessments (WAIS-IV), ensuring consistent methodology across subtypes. Subgroup analyses by hearing-loss configuration were not conducted because the small sample sizes in each group would have limited the power to detect significant differences. Therefore, analyzing the entire sample as a single group provides sufficient statistical power and preserves the internal consistency of the study design.

The inclusion criteria were: 18 years of age or older; severe or profound sensorineural hearing loss acquired after birth; native-level proficiency in German, as all assessment tools were administered in German; and completion of the WAIS-IV test before and after CI. The exclusion criteria included premature study withdrawal, inability to give informed consent, unwillingness to participate, and failure to use a cochlear implant.

### 2.2. Auditory Rehabilitation

All patients underwent outpatient cochlear implant rehabilitation at the tertiary clinic or affiliated centers. The rehabilitation lasted approximately 1.5 years per participant and comprised 20 sessions. These sessions focused on auditory training conducted by professional speech therapists and included speech processor fitting.

### 2.3. Assessment of Hearing Performance

#### 2.3.1. Freiburg Monosyllable Test

The Freiburg Monosyllable Test (FS), developed in 1953 [22], is a standard German speech-audiometry test [23]. The test includes 20-word lists, each with 20 monosyllabic nouns that may sound similar. The words are transmitted via loudspeakers, and the participant repeats what they recognize. The results are reported as a percentage of words recognized. For individuals with healthy hearing, 100% intelligibility is expected at a speech sound pressure level (SSP) of 50 dB or greater [24]. In this study, FS was performed at 65 dB SSP, without background noise, at the preoperative and 12-month postoperative time points.

During this study, speech intelligibility with hearing aids (when applicable) was assessed preoperatively using the Freiburg Monosyllabic Word Test presented at 65 dB in free-field conditions (loudspeaker placed at 0°). The contralateral ear was masked with white noise.

All patients (32) completed the FS before and after CI.

#### 2.3.2. Oldenburg Inventory (OI)

The Oldenburg Inventory (OI) was developed in 1991 to assess subjective hearing ability [25,26]. A 12-item shortened version was used, divided into three subdomains: hearing in quiet (5 items), hearing in noise (5 items), and directional hearing (*2* items). The response options range from “always” (5 points) to “never” (1 point). Subdomain and total scores are calculated from responses, with higher scores indicating better subjective hearing ability [25,26].

Twenty-five patients completed the OI before and after CI.

### 2.4. Cognitive Testing

Two cognitive functions, working memory and processing speed, were assessed using the Wechsler Adult Intelligence Scale—Fourth Edition (WAIS-IV), measuring the intellectual performance as described earlier by Knopke et al. [27]. Working memory (WM) was assessed using two tests: the Digit Span (DS) and the Arithmetic Reasoning (AR). In DS, patients repeat spoken digits in the same (forward), reverse (backward), and ascending (sequencing) order. In Arithmetic Reasoning, simple math problems were read aloud to the patient and then required to be solved. The problems became more difficult if the previous ones were answered correctly. Both tasks rely on auditory skills; therefore, examiners ensure participants understand the materials. According to the WAIS-IV manual, these tasks demand attention, auditory processing, cognitive flexibility, mental rotation, transformation, concentration, memory, and working memory capacity [28].

Processing speed (PS) was assessed with two timed subtests: Symbol Search (SyS) and Coding. In SyS, patients view a target symbol, then a row of symbols, and confirm if the target appears. In Coding, patients replicate symbols linked to numbers via a key. Testing PS involves concentration, visual memory, discrimination, flexibility, coordination, and scanning [28].

Scores were determined by the speed and accuracy with which a patient completed the tasks. WAIS-IV scoring procedures follow a standardized algorithm in which raw scores from each subtest are converted into age-normed scaled scores (mean = 10, SD = 3), as described in the WAIS-IV manual [28]. These scaled scores are then aggregated into index scores (mean = 100, SD = 15) based on factor-analytic structures that define the Working Memory Index (Digit Span, Arithmetic) and Processing Speed Index (Symbol Search, Coding). A higher number of correct responses or a larger proportion of pairs completed within the time limit indicates faster processing speed and stronger working memory.

All patients (32) completed cognitive testing before and after CI.

### 2.5. Patient Self-Report Questionnaires

#### 2.5.1. Nijmegen Cochlear Implant Questionnaire (NCIQ)

The NCIQ is a self-assessment questionnaire for health-related quality of life (HRQOL) in adult CI users [29]. It covers three areas—physical, psychological, and social functioning—divided into six subdomains: sound perception, speech and music perception, voice control, psychosocial values, social activity, and contacts. Each has ten questions, totaling 60. Answers are scored on a 5-point Likert scale, ranging from 0 to 100, and evaluated as previously described [29]. The total score is reported as a mean, with higher scores indicating better health-related quality of life.

Twenty-six patients completed NCIQ before and after CI.

#### 2.5.2. General Depression Scale—Long Form (ADS-L)

The ADS-L is used to evaluate depressive symptoms in individuals aged 12 and older [30]. The questionnaire comprises 20 items. The maximum achievable score is 60 points, with higher scores indicating more severe depressive symptoms. A score over 22 points is the threshold for the presence of depression [30,31].

#### 2.5.3. Tinnitus Questionnaire (TQ)

The TQ is used for subjective assessment of tinnitus-related distress [32]. The TQ comprises 52 items, grouped into the following categories: emotional distress (E), cognitive distress (C), intrusiveness of tinnitus (I), auditory difficulties related to tinnitus (A), sleep disturbances (Sl), and somatic complaints (SO). Psychological distress encompasses both emotional and cognitive distress. The total score is calculated as the sum of all subscales and ranges from 0 (no distress) to 84 points (maximum distress).

### 2.6. Statistical Tests

Statistical procedures were selected according to the distributional properties of the data, the measurement scales of the variables, and the modest sample size, which limits the applicability of parametric tests. Because several variables exhibited non-normal distributions and included ordinal psychometric measures, nonparametric methods were applied.

Pre–post differences were evaluated using the Wilcoxon matched-pairs signed-rank test, which is appropriate for dependent samples that do not satisfy parametric assumptions. The standardized Wilcoxon test statistic (Z) was calculated using Equation (1):

Z = (T − μT)/σT(1)

where T denotes the Wilcoxon test statistic, μT is the expected value under the null hypothesis, and σT is the corresponding standard deviation.

Effect sizes were derived from the standardized test statistic Z and the sample size N, following Cohen’s convention, using Equation (2):

r = Z/√N(2)

This effect-size index quantifies the magnitude of change independently of sample size and supports the interpretation of the practical relevance of findings. Thresholds for interpreting r were as follows: r < 0.10, no effect; 0.10–0.30, small effect; 0.30–0.50, medium effect; 0.50–0.70, large effect; r > 0.70, very large effect.

Associations between auditory, cognitive, and psychosocial variables were analyzed using Spearman’s rank correlation coefficient (ρ), which does not assume normality and is appropriate for ordinal variables and nonlinear relationships.

All calculations were performed using IBM SPSS Statistics version 29.0 (IBM Deutschland GmbH, Böblingen, Germany). Plots were created using BioRender (www.BioRender.com).

## 3. Results

### 3.1. The Auditory Abilities Measured with FS Improve After One Year of Auditory Rehabilitation with CI

**Figure 1:**
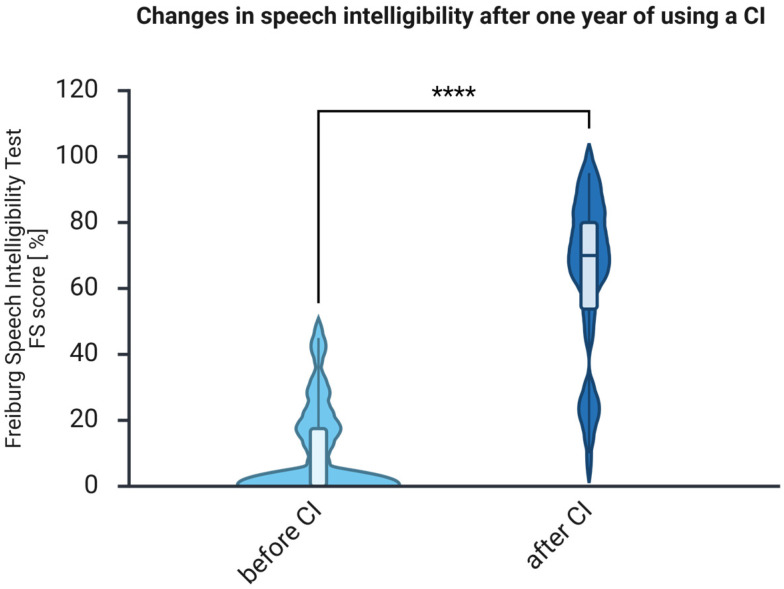
One year of auditory rehabilitation with CI significantly improves patients’ speech intelligibility. *n* = 32. Presented are violin plots illustrating medians and interquartile ranges (IQRs). The pre-post differences were calculated using the Wilcoxon matched-pairs test, ****, *p* < 0.001.

The auditory abilities measured in the affected ear using FS improved after CI (Figure 1) from a median of 0 (IQR 0–18.75) to 70.0 (IQR 50–80). The Wilcoxon matched-pairs test revealed that this improvement was statistically significant (Z = −4.864, *p* < 0.001, *r* = −0.61), indicating a large effect size. The improvement was noted in all patients.

### 3.2. Subjective Hearing Improves After One Year of Using CI

**Figure 2:**
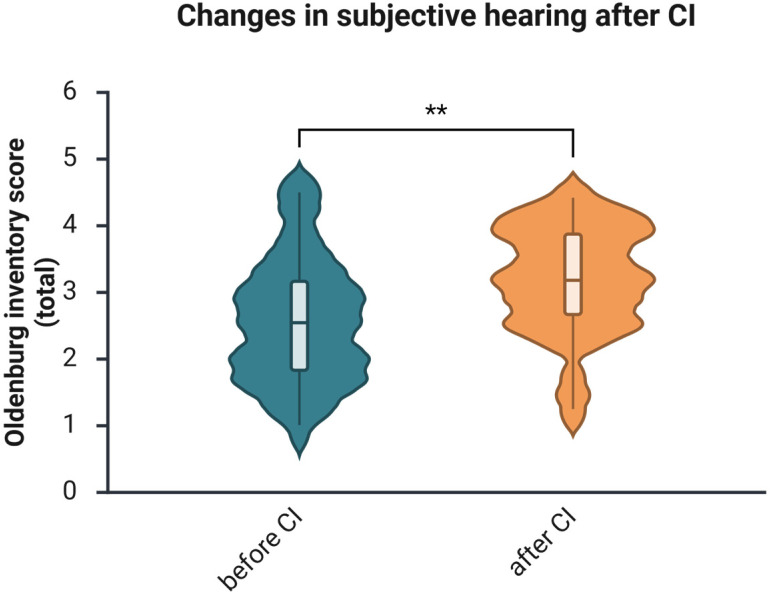
Changes in subjective hearing as per the total OI score after one year of auditory rehabilitation with CI. *n* = 25. Presented are violin plots illustrating medians and interquartile ranges (IQRs). The pre-post differences were calculated using the Wilcoxon matched-pairs test, **, *p* < 0.01.

Positive changes in hearing abilities were also documented by the OI. The subscales “hearing in quiet,” “hearing in noise,” and “sound localization” showed improvements after one year of using CI (Figure 2), as did the total score, which rose from a median of 2.55 to 3.18 (Z = −3.072, *p* = 0.002). The effect size was medium (*r* = −0.42).

### 3.3. The Working Memory Improves After One Year of Using CI

**Figure 3:**
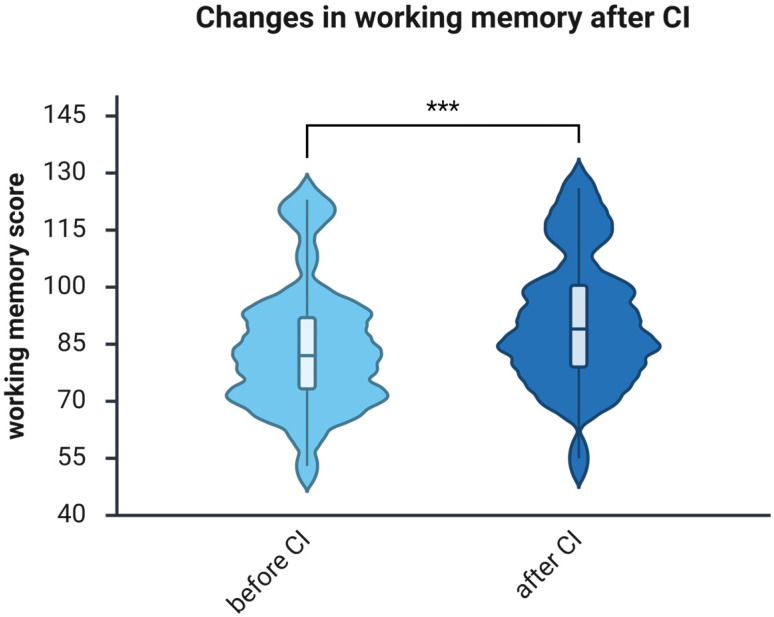
Working memory, as measured by the WAIS-IV, significantly improves 1 year after using CI. *n* = 32. Presented are violin plots illustrating medians and interquartile ranges (IQRs). The pre-post differences were calculated using the Wilcoxon matched-pairs test, ***, *p* < 0.001.

Before implantation, working memory had a median score of 82.00 points (IQR, 71.75–92.00; *n* = 32). One year after auditory rehabilitation with CI, the median increased to 89.00 points (IQR 79.00–101.50; *n* = 32). The Wilcoxon matched-pairs test revealed a significant improvement (Z = −4.090, *p* < 0.001; Figure 3), with a large effect size (*r* = −0.51).

### 3.4. The Processing Speed Improves After One Year of Using CI

**Figure 4:**
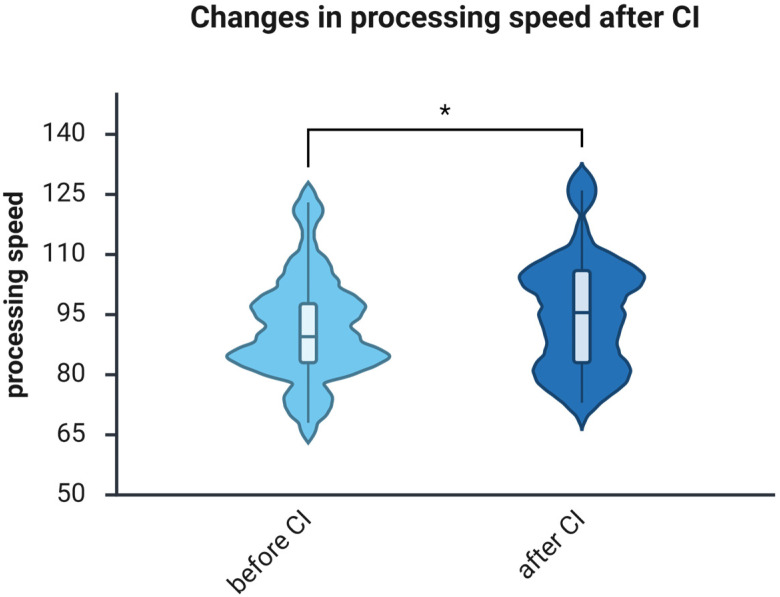
Processing speed, as measured with the WAIS-IV, significantly improves one year after using CI. *n* = 32. Presented are violin plots illustrating medians and interquartile ranges (IQRs). The pre-post differences were calculated using the Wilcoxon matched-pairs test, *, *p* < 0.05.

Processing speed had a median of 89.50 points before surgery (IQR, 83.00–99.25) and 95.50 points one year after implantation (IQR, 83.00–106.00). The Wilcoxon matched-pairs test indicated a significant improvement (Z = −2.533, *p* = 0.011); the effect size was medium (*r* = −0.32) (Figure 4).

### 3.5. The Health-Related Quality of Life Increases After One Year of Using CI

**Figure 5:**
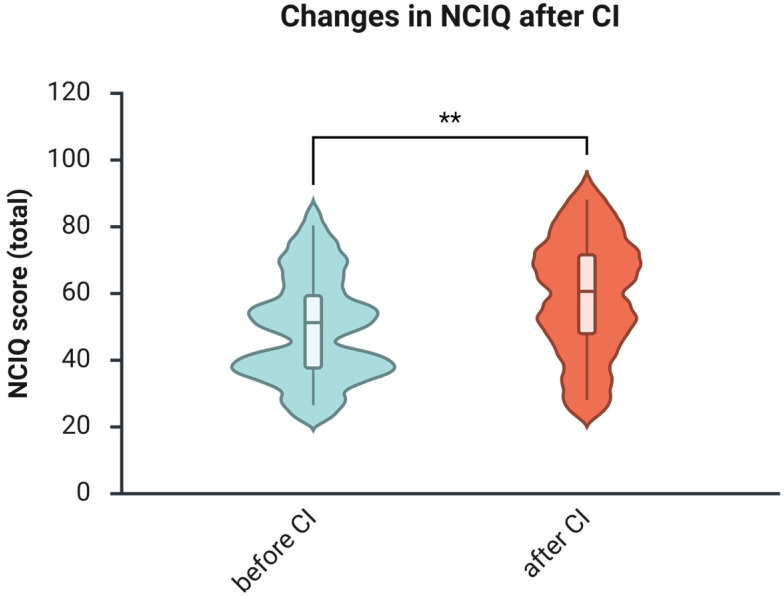
Health-related quality of life, as measured by the NCIQ, improves significantly after 1 year of CI use. *n* = 26. Presented are violin plots illustrating medians and interquartile ranges (IQRs). The pre-post differences were calculated using the Wilcoxon matched-pairs test, **, *p* < 0.01.

Subjective health-related quality of life, measured using the Nijmegen Cochlear Implant Questionnaire (NCIQ), showed postoperative improvements in the overall score (Figure 5). The Wilcoxon signed-rank test revealed a significant improvement in the total score from a median of 52.3 before CI to 60.6 one year after CI (Z = −3.899, *p* < 0.001), with a medium effect size (*r* = −0.49).

### 3.6. The Depressive Symptoms Do Not Change After One Year of Using CI

Of the 32 included patients, 25 completed the assessment of depressive symptoms before and after CI using the ADS-L, showing a reduction, with the median score decreasing from 21.00 to 16.00 points. However, the Wilcoxon signed-rank test did not indicate a significant change, with a Z-score of −1.703 and a non-significant *p*-value of 0.089.

### 3.7. The Tinnitus-Induced Distress Decreases After One Year of Using CI

Of the 32 included patients, 28 completed the TQ, allowing for the analysis of tinnitus-induced distress before and after implantation. Of them, 5 patients (15.6%) were tinnitus-free before implantation, and 8 patients (25%) were tinnitus-free after one year of CI use. One initially tinnitus-free patient reported tinnitus one year after implantation (TQ = 14, habituated/compensated tinnitus); the other four remained tinnitus-free.

**Figure 6:**
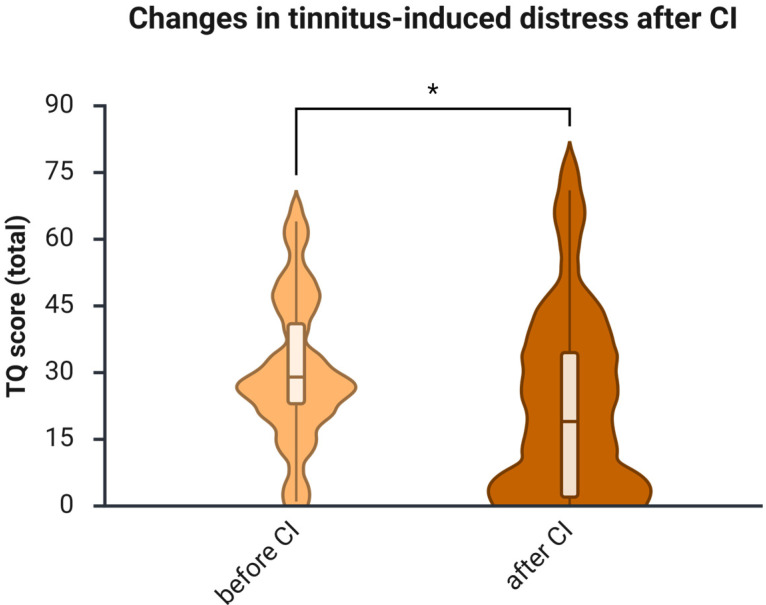
Tinnitus-induced distress significantly decreases after 1 year of CI use; *n* = 22. Presented are violin plots illustrating medians and interquartile ranges (IQRs). The pre-post differences were calculated using the Wilcoxon matched-pairs test, *, *p* < 0.05.

Significant improvements in tinnitus-related distress were observed (Figure 6). Before CI, 23 patients reported having tinnitus. Of these, 22 completed the TQ one year after using CI. In this group, the median TQ score decreased from 25 before CI to 21 one year after implantation, as indicated by the Wilcoxon test (Z = −2.209, *p* = 0.027), with a medium effect size (*r* = −0.333).

### 3.8. Correlations Between Variables Before and After CI

To determine the strength and direction of relationships between pairs of variables, Spearman’s rank correlation was used, with the primary goal of identifying associations between the cognitive variables (WM or PS) and other variables. Before CI, WM and PS were strongly correlated (Table 3). Additionally, there was a medium correlation between education level and WM, but not PS. A strong positive correlation was also found between OI and NCIQ, and a medium negative correlation between TQ and NCIQ.

One year after CI, a strong positive correlation between WM and PS, as well as a medium correlation between WM and education level, remained (Table 4). The strong correlation between NCIQ and OI persisted, and the correlation between NCIQ and TQ became strong. One medium negative correlation emerged between WM and TQ.

Three new significant positive correlations emerged for PS: the first linking it to NCIQ (strong correlation), the second to OI (medium correlation), and the third to education level (medium correlation).

Furthermore, a strong positive correlation between OI and NCIQ, as well as a strong negative correlation between TQ and NCIQ, persisted. In contrast, new negative correlations were identified between NCIQ and ADSL (medium) and between TQ and OI (strong). Additionally, a significant positive correlation was observed between ADSL and TQ.

## 4. Discussion

The current study investigates auditory and cognitive changes in a sample of middle-aged adults with hearing loss who underwent CI-based auditory rehabilitation. Additionally, most patients were evaluated for health-related quality of life, tinnitus-related distress, and depressive symptoms. Speech intelligibility significantly improved after implantation, as indicated by the FS test (Figure 1) and the patient self-report questionnaire (OI; Figure 2), consistent with several earlier reports [34,35,36]. Furthermore, at 1 year post-implantation, improvements in health-related quality of life (see Figure 5) and reductions in tinnitus-related distress (see Figure 6) were observed, consistent with prior research findings [37]. There was also a reduction in depressive symptoms, although it was not statistically significant. Since the occurrence of depressive symptoms in adults varies with age, economic conditions, and political factors—and has appeared to rise in recent years [38]—it is probable that some of these influences, such as the COVID-19 pandemic in 2020 and 2021, impacted the patients and may have obscured the positive effects of CI on depressive symptoms reported by earlier studies [39,40].

The main goal of the present study was to determine if CI influences cognitive abilities in the middle-aged hearing-impaired patients. Using the WAIS-IV, two cognitive skills—WM and PS—were assessed before and after 1 year of auditory rehabilitation with a CI, revealing significant improvements in both WM (Figure 3) and PS (Figure 4). The effect size for improvements in WM was large, whereas that for PS was medium. Two additional studies were previously conducted with a goal similar to this one. In the first study, Mosnier et al. tested the PS using only one test from WAIS-IV, namely Digit Coding, instead of two as in the present study, and observed improvement only in the “young old” (60–64), not in the “middle old” (65–75), or “old old” (75+) groups [14]. Their findings are consistent with existing observations; however, the “young old” group (60–64) only partially overlaps with the age range sampled in this study (32–65). Furthermore, Mosnier et al. adjusted their calculations based on patients’ education level and age. The current correlation analysis showed no association between WAIS-IV scores and age (Table 3 and Table 4). Interestingly, correlations between education level and WM were observed in the present study both before and after CI, whereas correlations between education level and PS were observed only after CI. Correlation analyses revealed stronger associations among the auditory, cognitive, and psychosocial domains following cochlear implantation. Processing speed positively correlated with quality of life (NCIQ; ρ = 0.510, *p* = 0.006), subjective hearing (OI; ρ = 0.400, *p* = 0.039), and education (ρ = 0.378, *p* = 0.040). Working memory was negatively associated with tinnitus distress (TQ; ρ = –0.459, *p* = 0.021). These suggest that better auditory access can enhance cognitive efficiency, reduce effort, and support psychosocial functioning. Post-implantation, cognitive performance in middle-aged adults appears increasingly linked with auditory and emotional processes as input improves.

In the second study, which had a similar aim to the current one, Calvino et al. examined cognitive changes in patients under 60 and compared them with those in patients over 60 [15]. However, different outcome measures were used than in the present study, namely the Assessment of Neuropsychological Status for Hearing-Impaired Individuals (RBANS-H). Calvino et al.’s results indicated a significant improvement in the total RBANS-H score in both age groups one year after CI. RBANS-H evaluates 5 cognitive domains: immediate memory, delayed memory, attention, language, and visuospatial abilities. Among them, the scores in the RBANS domain “attention” were found by others to strongly correlate with PS results on the WAIS-IV [41], allowing for comparative analysis of this cognitive variable. Interestingly, Calvino et al. found no enhancement in “attention” among patients aged 60 or younger, which differs from the results of the present study. One explanation for this discrepancy is the age difference between the study groups. Calvino et al.’s study included significantly younger individuals (mean age 48.7 ± 8.3), whereas the present study included individuals with a mean age of 52.4 ± 9. Processing speed usually decreases between ages 34 and 44 in a healthy lifespan [42]; therefore, expecting improvements in individuals younger than that is unlikely. The decline in WM and PS is a common characteristic not only of normal cognitive aging [43] but also among individuals with mild cognitive impairment and early Alzheimer’s disease [44].

Comparing this study’s outcomes with other research is challenging for two main reasons: differences in outcome measures and variations in the age composition of the samples. The WAIS-IV is rarely used to assess cognition in CI candidates [45]. Additionally, most research either focuses on patients aged 60 or older or on groups that include younger, older, and elderly participants together [14,45]. Interestingly, a recent meta-analysis found that, across age groups, only about half of the studies reported cognitive improvements after cochlear implantation [39]. This variation is often attributed to differences in the cognitive domains examined and the variety of outcome measurement tools used. Overall, this study found cognitive improvements following CI-based auditory rehabilitation in middle-aged hearing-impaired patients, consistent with the findings of Mesnier et al. and Calvino et al. [14,15].

In recent years, the role of hearing loss in cognitive decline has attracted increasing attention, and several hypotheses have been proposed to explain this link. The first, the “common cause hypothesis,” suggests a shared pathological mechanism, such as vascular disease, that contributes to both hearing loss and dementia [46]. The second “sensory deprivation/brain structural changes” hypothesis suggests that the absence of auditory input leads to both structural and functional brain changes, including a reduction in gray matter volume, which directly contributes to cognitive decline [46,47]. The third hypothesis, called the “cognitive load” hypothesis, proposes that hearing loss increases mental effort, thereby reducing the resources available for other cognitive processes [48,49]. Finally, the fourth “information degradation hypothesis” suggests that hearing impairment reduces the quality of sensory information delivered to the brain [46,49,50,51]. The above hypotheses are probably not mutually exclusive, and it is possible that multiple, or even all, of the proposed mechanisms could play a role in the development or progression of cognitive decline.

The current research did not focus on organic changes, such as vascular pathology or brain structural changes; therefore, the presented results neither support nor refute the first two hypotheses. The last two hypotheses suggest that hearing loss may increase cognitive load, and as sensory information quality improves, cognitive load decreases, freeing up more mental resources. This study supports these hypotheses by showing that enhancing previously impaired hearing abilities through auditory rehabilitation using CI is associated with improved cognitive skills. Importantly, beyond various studies focusing on older hearing-impaired adults, this research demonstrates that cognitive improvements are also observed in middle-aged hearing-impaired adults. This group has been identified by others as especially vulnerable to future cognitive decline and as a group in which auditory rehabilitation might help modify these changes [10,11,12].

Interestingly, evidence indicates that, in healthy older adults, auditory training alone and in combination with cognitive training can markedly improve functional brain connectivity [52]. Moreover, cognitive tasks such as the logical memory and digit cancellation tests tend to improve following combined cognitive-auditory training [52]. These observations further support the hypothesis of a link between the auditory system and cognition.

This study is not without limitations. The first significant drawback is that the subjects differed in terms of the type of hearing loss (DSD, AHL, SSD). These differences are known to lead to differences in cerebral cortex activation, characterized by metabolic asymmetry in PET imaging for SSD and AHL patients [19,21] and a general hypometabolism in DSD patients [53]. Such differences could affect the outcome measures used in the present study (WM and PS). However, cognitive skills were shown to be affected by all types of hearing impairment, including SSD [20] and AHL [54]. The second limitation is that it only examines a single time point after CI. It is essential to determine whether the cognitive gains are sustained and for what duration. Finally, although all patients in the sample underwent audiometric testing and participated in the WAIS-IV, not all completed the remaining questionnaires, which is also a pitfall.

Despite these limitations, the current findings strongly support the conclusion that cochlear implantation can impact cognitive performance in middle-aged adults. The notable improvements in working memory and processing speed seen in this group are not only statistically significant but also align with theoretical models suggesting that better auditory input reduces cognitive load and enhances the efficiency of central information processing. The presence of postoperative links between auditory measures (OI, NCIQ) and cognitive outcomes suggests that better auditory access could enhance overall cognitive engagement in daily activities. Significantly, the cognitive enhancements occurred despite no notable changes in depressive symptoms, suggesting that these effects are not linked to mood improvements. Overall, these findings indicate that auditory rehabilitation plays a significant role in supporting cognitive function and, consequently, that CI may affect the course of cognitive aging in this population.

The future outlook for projects similar to the one discussed in this paper includes conducting a comparative analysis of a large patient sample based on hearing loss type and duration. Additionally, long-term studies spanning several years will be an essential component of future research.

## 5. Conclusions

This study aimed to evaluate potential cognitive changes after one year of CI hearing rehabilitation in a middle-aged group. This age group is particularly vulnerable to developing dementia later in life when experiencing untreated hearing loss. The results indicate that, alongside auditory and hearing-related factors, cognitive functions such as working memory and processing speed also improve following implantation in these middle-aged patients. This study’s findings support recommending cochlear implants for middle-aged patients with hearing loss who do not benefit from hearing aids, helping them restore hearing and potentially modifying the risk of cognitive decline.

## Figures and Tables

**Table 1:** Comparative overview of prior studies, their limitations, identified research gaps, and how the current study addresses them.

Study	Population/Age, Range	Cognitive Measure Used	Key Findings	Methodological Weaknesses	Research Gaps	How the Present Study Addresses the Gaps
Mosnier et al. (2024) [14]	100 CI users; 60–64, 65–75, ≥75	MMSE, TMT-B, Digit Symbol Coding, TUG	Cognitive improvement only in the 60–64 group; auditory gains	No adults < 60 included	No evaluation of WMI or PSI with full WAIS-IV Only one WAIS-IV subtest was used (Digit Coding) Midlife cognitive effects were not assessed	Focuses on midlife adults; uses full WAIS-IV WMI and PSI. Provides midlife-specific cognitive data with standardized measures.
Calvino et al. (2022) [15]	28 CI users ≤ 60 and ≥61	RBANS-H (attention, memory, visuospatial)	Cognitive improvement in both age groups	Broad age categories without specific midlife delineation	No targeted analysis of midlife adults RBANS-H does not isolate WM or PS	Specifically includes midlife adults Applies WAIS-IV, the gold standard for WMI and PSI

MMSE, the Mini-Mental State Examination; RBANS-H: Repeatable Battery for the Assessment of Neuropsychological Status—Hearing Impaired Version; TMT-B, Trail Making Test—Part B; TUG, Timed Up and Go Test; WAIS-IV, the Wechsler Adult Intelligence Scale—Fourth Edition; PS(I), processing speed (index); WM(I), working memory (index).

**Table 2:** Patient characteristics before CI.

Parameter	Number of Patients or a Mean with SD
Age (years)	52.4 (9.6)
Sex	14 women and 18 men
Freiburg Monosyllabic Test result on the ear scheduled for implantation	9.2 (13.1)
Type of hearing loss	
AHL (asymmetric hearing loss)	7
SSD (single-sided or unilateral deafness)	11
DSD (double-sided or bilateral deafness)	14
Cause of hearing loss	
acoustic trauma	1
an accident	1
autoimmune disease	1
cholesteatoma	1
Ménière’s disease	1
middle ear infection	2
noise-induced hearing loss	1
sudden hearing loss	8
surgery	1
unknown	9
Highest education level	
no education	1
primary school	5
comprehensive school	17
grammar school	2
technical college	3
university	2
no data	2

**Table 3:** Spearman’s correlation analysis before CI.

			Working Memory Before CI	Processing Speed Before CI	NCIQ Total	ADSL	OI Total	TQ Total	Level of Education
Spearman’s rho	**processing speed before CI**	Correlation Coefficient	0.533 **						
		Sig. (2-tailed)	0.002						
		*n*	32						
	**NCIQ total**	Correlation Coefficient	0.208	0.048					
		Sig. (2-tailed)	0.289	0.809					
		*n*	28	28					
	**ADSL**	Correlation Coefficient	−0.029	−0.335	−0.163				
		Sig. (2-tailed)	0.888	0.087	0.416				
		*n*	27	27	27				
	**OI total**	Correlation Coefficient	−0.004	0.037	0.781 **	−0.066			
		Sig. (2-tailed)	0.984	0.854	<0.001	0.743			
		*n*	27	27	27	27			
	**TQ total**	Correlation Coefficient	−0.059	0.059	−0.383 *	0.234	−0.137		
		Sig. (2-tailed)	0.772	0.770	0.049	0.251	0.505		
		*n*	27	27	27	26	26		
	**level of education**	Correlation Coefficient	0.452 *	0.320	0.230	−0.004	0.315	−0.016	
		Sig. (2-tailed)	0.012	0.084	0.239	0.985	0.109	0.938	
		*n*	30	30	28	27	27	27	
	**age**	Correlation Coefficient	−0.031	0.035	−0.088	−0.059	−0.036	0.184	−0.238
		Sig. (2-tailed)	0.864	0.851	0.658	0.770	0.858	0.359	0.206
		*n*	32	32	28	27	27	27	30

**. Correlation is significant at the 0.01 level (2-tailed); *. Correlation is significant at the 0.05 level (2-tailed); *n*, sample size; Spearman correlations were interpreted according to Cohen’s guidelines: small (≈0.10), medium (≈0.30), large (≈0.50) [33]. Gray shading indicates correlations between cognitive variables and other measured parameters.

**Table 4:** Spearman’s correlation analysis after CI.

			Working Memory After CI	Processing Speed After CI	NCIQ Total	ADSL	OI Total	TQ Total	Level of Education
Spearman’s rho	**processing speed after CI**	Correlation Coefficient	0.516 **						
		Sig. (2-tailed)	0.002						
		*n*	32						
	**NCIQ total**	Correlation Coefficient	0.361	0.510 **					
		Sig. (2-tailed)	0.059	0.006					
		*n*	28	28					
	**ADSL**	Correlation Coefficient	−0.171	−0.261	−0.476 *				
		Sig. (2-tailed)	0.394	0.189	0.012				
		*n*	27	27	27				
	**OI total**	Correlation Coefficient	0.279	0.400 *	0.858 **	−0.312			
		Sig. (2-tailed)	0.159	0.039	<0.001	0.114			
		*n*	27	27	27	27			
	**TQ total**	Correlation Coefficient	−0.459 *	−0.381	−0.720 **	0.591 **	−0.624 **		
		Sig. (2-tailed)	0.021	0.060	<0.001	0.002	0.001		
		*n*	25	25	25	24	24		
	**level of education**	Correlation Coefficient	0.496 **	0.378 *	0.296	0.005	0.280	−0.161	
		Sig. (2-tailed)	0.005	0.040	0.143	0.981	0.165	0.464	
		*n*	30	30	26	26	26	23	
	**age**	Correlation Coefficient	−0.019	−0.035	−0.149	0.016	−0.049	−0.104	−0.238
		Sig. (2-tailed)	0.916	0.848	0.448	0.935	0.806	0.620	0.206
		*n*	32	32	28	27	27	25	30

**. Correlation is significant at the 0.01 level (2-tailed); *. Correlation is significant at the 0.05 level (2-tailed); *n*, sample size; Spearman correlations were interpreted according to Cohen’s guidelines: small (≈0.10), medium (≈0.30), large (≈0.50) [33]. Gray shading indicates correlations between cognitive variables and other measured parameters.

## Data Availability

Due to the need to protect patient confidentiality, data are available from the corresponding author upon reasonable request.

## Abbreviations

The following abbreviations are used in this manuscript:

ADS-L	General Depression Scale (long version questionnaire)
AHL	Asymmetric Hearing Loss
CI	Cochlear Implant
FS	Freiburg Monosyllable Test (speech Intelligibility test)
GAD-7	Generalized Anxiety Disorder 7
NCIQ	Nijmegen Cochlear Implant Questionnaire (health-related quality of life)
OI	Oldenburg Inventory
PSQ	Perceived Stress Questionnaire
TQ	Tinnitus Questionnaire
